# Identification of biomarkers for development of end-stage kidney disease in chronic kidney disease by metabolomic profiling

**DOI:** 10.1038/srep26138

**Published:** 2016-05-18

**Authors:** Tomonori Kimura, Keiko Yasuda, Ryohei Yamamoto, Tomoyoshi Soga, Hiromi Rakugi, Terumasa Hayashi, Yoshitaka Isaka

**Affiliations:** 1 Department of Nephrology, Osaka University Graduate School of Medicine, Box B6, 2-2 Yamada-oka, Suita, Osaka, 565-0871, Japan; 2 Institute for Advanced Biosciences, Keio University, 246-2, Mizukami, Kakuganji, Tsuruoka, Yamagata 997-0052, Japan; 3 Department of Geriatric and General Medicine, Osaka University Graduate School of Medicine, Box B6, 2-2 Yamada-oka, Suita, Osaka, 565-0871, Japan; 4 Department of Kidney Disease and Hypertension, Osaka General Medical Centre, 3-1-56, Bandaihigashi, Sumiyoshi-ku, Osaka, 558-8558, Japan; 5 Department of Nephrology, Rinku General Medical Centre, Izumisano Municipal Hospital, 2-23 Rinku-Orai Kita, Izumisano, Osaka 598-8577, Japan

## Abstract

A critical issue in the management of chronic kidney disease (CKD) is to prevent patients from the progression to end-stage kidney disease (ESKD), however, there is only limited number of biomarkers for the discrimination of the high-risk CKD patients. We aimed to identify the metabolites which possess the ability to predict the earlier kidney deterioration. We performed capillary electrophoresis and liquid chromatography mass spectrometry (CE-MS)-based metabolic profiling in a prospective cohort, which consisted of referred 112 CKD patients with median follow-up period of 4.4 years. The association between the levels of candidate metabolites and the outcomes (progression to ESKD alone or in combination with death before ESKD) were assessed by multivariate Cox proportional hazard models after adjusting for the baseline covariates. A total of 218 metabolites were detected in the plasma of CKD patients. We identified 16 metabolites which have predictive values for the composite outcome: The risk for composite outcome was elevated from 2.0- to 8.0-fold in those with higher levels of 16 plasma metabolites. Our results suggest that the measurement of these metabolites may facilitate CKD management by predicting the risk of progression to ESKD.

Chronic kidney disease (CKD) is a global health problem^1^. CKD patients are widely prevalent, and the number of end-stage kidney disease (ESKD) patients is still increasing. ESKD patients requires cost-prohibitive kidney replacement therapy^2^. Moreover, CKD patients are highly vulnerable, and the risk of cardiovascular events and death increases with the progression of CKD stages^3^,^4^,^5^,^6^. Thus, it is critical to predict their risk for the progression to ESKD in CKD patients to avoid these unfavorable situations.

Taking the fact that kidney is one of metabolically-active organs^7^,^8^, the metabolic profiling of CKD patients is a promising method to identify new biomarkers for the prognoses of CKD patients. Recent cross-sectional studies have demonstrated the correlations between kidney function and the levels of certain metabolites in CKD patients^9^,^10^,^11^,^12^,^13^. The presence or altered levels of certain metabolites was also suggested in ESKD patients^14^. Population-based studies also demonstrated that the levels of some metabolites were associated with the incidence of CKD^15^,^16^ or with the worsening of kidney function, i.e., the decrease in estimated glomerular filtration ratio (eGFR, a calculated product of creatinine, sex, and age)^17^,^18^. A nested-cohort study in diabetic patients with early stages of CKD (mainly stage 1 and 2) showed a specific metabolomic profile in those who progressed to ESKD within an observational period^19^. The importance of metabolic profiling and the aberrance of metabolism in CKD patients have been emphasized^20^, however, the number of prospective metabolomic studies is limited thus far. Thus, whether certain metabolic changes could predict rapid progression of ESKD in referred CKD patients is largely unknown.

We conducted capillary electrophoresis and liquid chromatography mass spectrometry (CE-MS)-based metabolic profiling^21^,^22^ in a prospective longitudinal cohort, which was composed of referred CKD patients who were not on dialysis at entry. We aimed to prove that the levels of a subset of blood metabolites would predict the further worsening of kidney function, with the goal to raise the possibility that the measurements of these metabolites will facilitate the management of referred CKD patients.

## Results

We performed a metabolic profiling from a prospective cohort, which consisted of referred CKD patients. Data were generated from 112 participants with median follow-up period of 4.4 years (IQR, 2.4–5.5). The baseline characteristic is shown in Table 1. None of the participants were lost to follow-up. Among these patients, 61 started kidney replacement therapy and 17 died, including 4 who died before initiating kidney replacement therapy.

Metabolomic analyses were performed using the plasma samples of this cohort. Out of 579 metabolites in the library, a total of 218 metabolites were detected in the plasma of CKD patients. Among them, 129 metabolites, including creatinine and urea nitrogen, were above the detection limits in at least 40% of study subjects and were subjected to the rest of the analyses. The levels of 73 out of 129 metabolites were significantly correlated with eGFR (Figs S1–3): the increased levels of urea and creatinine, which represent kidney function, showed the strongest correlation with the decreased level of eGFR, whereas the levels of the rest of metabolites variably, but less strongly, correlated.

**Figure 1:**
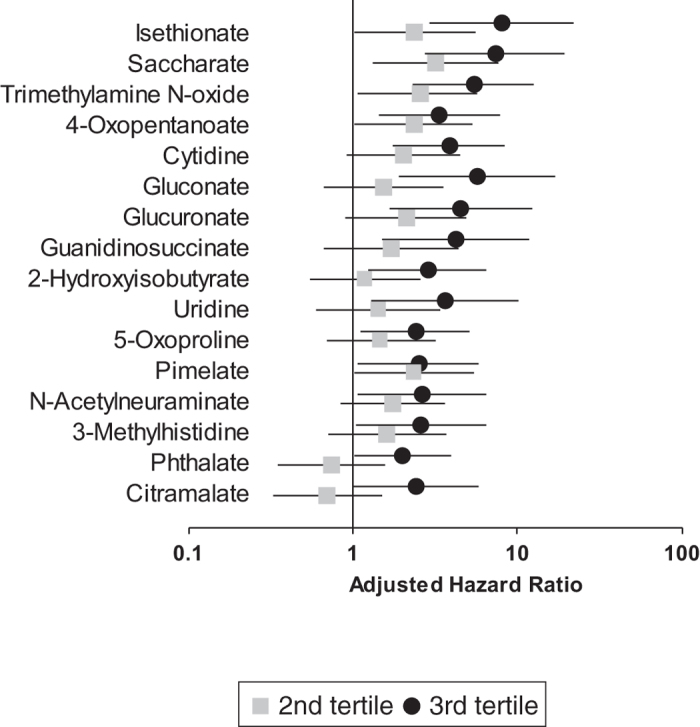
Forest plot of adjusted hazard ratio of each tertile as categorical variable for composite outcome. First tertile was used as reference.

The levels of metabolites were divided into three tertiles and were subjected to the Cox regression analyses. The Holm-corrected multiple testing revealed that the levels of 26 out of 127 metabolites (creatinine and urea were excluded) had predictive values on the composite outcome (a combination of end-stage kidney disease requiring replacement therapy and all-cause of death, Table S1). These metabolites were detected in more than 80% of patients except for 2-hydroxyisobutyrate (67.5%), cytidine (56.4%), and 3-hydroxy-3-methylglutarate (53.0%). We fitted multiple Cox regression analyses to assess the predictive value of the levels of these metabolites by adjusting for eGFR, the level of urinary protein, and other clinical covariates such as the presence of diabetes. After adjustment for the clinical covariates, the levels of 16 out of 26 metabolites remained as significant predictors of the composite outcome when tertiles were analyzed as continuous variables (Table 2). The results were largely similar when analyzed for ESKD (Table S2). Patients in the top tertile of these metabolites had 2.0- to 8.0- fold higher adjusted hazard ratios for the composite outcomes, compared with those in the lowest tertile (Fig. 1 and Tables 2 and S2).

**Figure 2:**
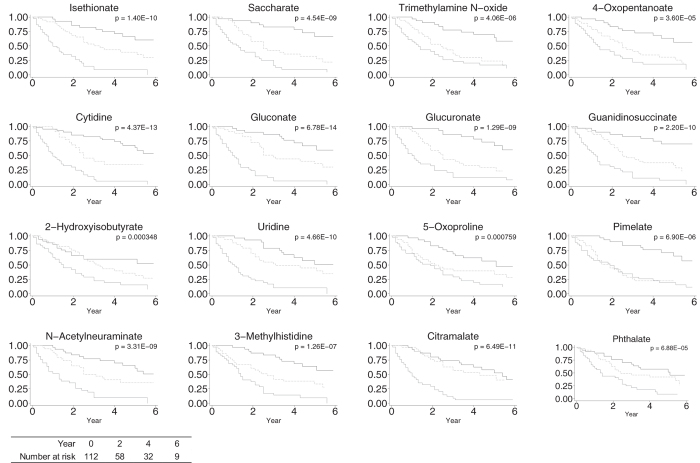
Kaplan-Meier survival curves of selected metabolites for composite outcome. Patients with first (thick line), second (dotted line), and third (thin gray line) tertile of levels of metabolites were subjected to these analyses.

Kaplan-Meier curve analyses demonstrated that patients with the higher levels of 58 metabolites including creatinine and urea, and the lower levels of 4 metabolites, more frequently reached the composite outcome (Figs 2 and S4). Among them, 16 metabolites identified in the analysis above robustly divided the likelihood of reaching the composite outcome (Fig. 2). Because not a small fraction of this cohort is diabetic patients, we stratified the analyses by its presence (Table S3 and Fig. S5). The levels of some metabolites showed good predictive values specifically in the presence (4-oxopentanoate, glucoronate, 2-hydroxyisobutyrate, 5-oxoproline, pimelate, N-acetylneuraminate, 3-methylhistidine, phthalate, trp, hippurate, and 3-hydroxy-3-methylglutarate) or absence (citramalate and 2,3-pyrinedicarboxylate) of diabetes, although the levels of some showed the predictive values regardless of the presence or the absence of diabetes (isethionate, saccharate, trimethylamine N-oxide, cytidine, gluconate, guanidinosuccinate, and uridine).

## Discussion

By applying CE-MS-based metabolic profiling, we identified 16 prognostic metabolites in CKD patients. The risk of developing ESKD was elevated from 2.0- to 8.0-fold in those with higher or lower levels of plasma metabolites. Although the levels of some of the metabolites identified in this study correlated with eGFR, these metabolites are associated with kidney deterioration even when they were adjusted for the baseline kidney function. These findings may support the notion that measurement of these metabolites in CKD patients would be a useful tool to assess the risk of progression to ESKD.

The number of studies using metabolomics in CKD studies as a tool is increasing, which strongly suggests the growing interests of this field^9^,^10^,^11^,^12^,^14^,^15^,^16^,^17^,^18^,^19^. In their first application of metabolomics on CKD study, Toyohara *et al.* demonstrated correlations between the levels of 64 plasma metabolites and that of eGFR in CKD patients^9^. Rhee *et al.* showed that the levels of 49 metabolites were altered in ESKD patients on hemodialysis compared with age-matched control^14^. Shah *et al.* showed significant differences in the levels of some plasma metabolites among various stages of CKD 2–4^10^. Hirayama *et al.* identified some metabolites whose levels were associated with the presence of nephropathy (assessed by the presence of macroalbuminuria) in diabetic patients^11^, while Sharma *et al.* demonstrated that the levels of 12 metabolites were associated with the presence of diabetes in CKD^12^. By nested-cohort study, Niewczas *et al.* demonstrated that the levels of some plasma metabolites had significant odds ratios for the incidence of ESKD during the observational period in diabetes patients (CKD stage 1–2, 78%; stage 3, 22%)^19^. In population-based studies, Rhee *et al.* and Yu *et al.* demonstrated that a subset of blood metabolites in study participants (eGFR ≥60 mL/min/1.73 m^2^) were associated with the onset of incident CKD (defined by eGFR of <60)^15^,^16^. Geok *et al.* demonstated that the levels or the combined ratios of some serum metabolites were associated with the decreased ratio of eGFR^17^,^18^. The present study added the important prognostic notion that patients with advanced CKD stages have particular metabolomic profiles that are associated with worse prognoses.

The 16 metabolites identified in the present cohort are located on widely variable metabolic pathways. These pathways include nucleotides (cytidine and uridine), glycolysis (gluconate [also known as gluconic acid] and saccharate [glucaric acid]), amino acids (guanidinosuccinate [guanidinosuccinic acid] and 3-methylhistidine), amino sugar (N-acetylneuramine [N-acetylneuraminic acid] and glucoronate [glucuronic acid]), biotin (pimelate [heptanedioic acid]), glutathione (5-oxoproline [pyroglutamic acid]), and taurine (isethionate [2-hydroxyethanesulfonate]). In addition, the origins or pathways of some metabolites are unidentified (4-Oxopentanoate [levulinic acid, 4-ketovalerate], trimethylamine N-oxide [TMAO], citramalate, 2-hydroxyisobutyrate [alpha-hydroxyisobutyric acid, acetonate], and phthalate [Alizarinate, Naphthalinate]). These metabolites might be originated from extracorporeal metabolisms such as gut flora, dietary, and environmental chemicals, whose importance in CKD patients are rediscovered recently^23^. The presence of broad pathways behind the identified metabolites in our study may reflect the complicated process of kidney deterioration^24^.

The association with kidney function have been demonstrated in some of metabolites identified in this study. Guanidinosuccinate, trimethylamine N-oxide, 3-methylhistidine^25^, cytidine^26^, and uridine^27^ are ones of the earliest uremic toxins whose toxicity are identified^28^,^29^. Trimethylamine N-oxide is also associated with onset of CKD^15^. Both cytidine and uridine are pyrimidine-derived nucleosides, and the level of cytidine is known to decrease in kidney from drug-induced kidney injury model rats^30^. Guanidinosuccinate is an acetyl derivative of the amino sugar neuraminic acid, whereas N-acetylneuramine, another metabolite identified in this study, is a major component of glycoconjugates, including glycoproteins which resides on cellular membranes to mediate several cellular functions. The fact that both guanidinosuccinate and N-acetylneuramine were identified as predictors for kidney deterioration in this study may suggest the potential roles of glycomodulation in the progression of kidney diseases. The fact that the level of 3-Methylhistidine, a component of actin and myosin, is associated with muscle protein breakdown^31^ may reflect muscle-wasting in kidney diseases^32^. 5-Oxoproline is known to increase its level in response to worsened kidney function^9^,^33^. 5-oxoproline is involved in the metabolism of glutathione, a major antioxidant, in both synthetic and degradative pathways. While our study is in revision, Yu *et al.* reported that lower levels 5-oxoproline was associated with incidence of CKD^16^, whereas our data suggested that its higher level was a risk for earlier kidney deterioration. The regulation of 5-oxoproline seems to be complicated and its level should be understood based on their kidney function and other underlying comorbidities. Interestingly, the levels of some metabolites have the potentials to predict prognosis in specific patients (i.e., in the presence or absence of diabetes). Whether these metabolites may reflect prognosis synergically or through specific association with certain underlying pathophysiology needs to be studied.

This study has some limitations that need to be addressed in further studies. Although the present study was strengthened by the longitudinal observation, the limitations exist on relatively small number of the cohort and the dependence only on the baseline characteristics. We performed power analyses using a type I error of 5% and 80% power. These analyses revealed that 19 or 65 patients would be needed for the median (3.62 for uridine) or lowest hazard ratios (2.01 for phthalate) of the selected metabolites in the prediction set, respectively. Therefore, we had enough sample size to determine the association between composite outcomes and these metabolites, though the effects of unselected metabolites might be underestimated. This is especially the case in the presence of statistical processes of multiple comparison or adjustments. The correlations between the metabolites identified in this study and eGFR also complicated the results. Although our results were achieved by adjusting for eGFR, we could not completely rule out residual confounding due to kidney function. We considered that the levels of these metabolites in CKD patients may not be simple reflections of the decreased level of eGFR based on the following observations^34^,^35^. First, the Spearman correlation coefficients for the selected metabolites with eGFR were not always the highest ones as shown in Fig. S2. Second, one metabolite (phthalate) was not significantly correlated with eGFR. Third, the coefficients of most of the metabolites were lower than that of hemoglobin (r = 0.58), which is used as a prognostic factor of CKD patients. Therefore, the metabolic profile of CKD patients identified in the present study may rather provide additional information for the progression of kidney disease. The importance of the identified metabolites and the variance in the concentrations of these metabolites need to be validated in future studies. Statistical multiple-comparison might have obscured the true relationship. Finally, we have no mechanistic data to demonstrate the reasons for the changes of the levels of these metabolites. Further mechanistic and causal studies together with validation studies will support our comprehensiveness and clinical application.

In summary, 16 metabolites were related to higher risk of the deterioration of kidney function in advanced CKD patients. Our studied population is based on referred CKD patients, who need immediate and impeding improvement of medical care. Measurement of the metabolites identified herein may facilitate our management of advanced CKD patients through the additional information on the clinical parameters, such as eGFR and urinary protein level. We propose that the metabolomic profiling of CKD patients will potentiate us to perform tailored management of CKD.

## Methods

### Study population and samples

We prospectively enrolled 118 consecutive patients with CKD stages 3, 4 and 5, who were not on dialysis, from a single nephrology department at Rinku General Medical Centre between August 2005 and January 2009. Baseline blood samples from patients after an overnight fast were collected. Patients with insufficient blood samples were excluded beforehand. This study was approved by the institutional ethics committees of Rinku General Medical Centre and Osaka General Medical Centre, and was adherent to the Declaration of Helsinki. All patients provided written informed consent to participate in the study.

Baseline inclusion criteria included age less than 90 years, no complication of malignancy, and no active infection. Patients with incomplete baseline data (n = 2) and started kidney replacement therapy within 1 month after the enrollment (n = 4) were excluded. Final analysis covered the remaining 112 patients.

Kidney function was evaluated from baseline data at the first visit to our outpatient clinic using the estimated glomerular filtration rate (eGFR) based on the equation for Japanese population^36^. The formula is as follows: eGFR = 194 × serum creatinine (SCr)^−1.094^ × age^−0.287^, where age is in years, SCr is in mg/dL and the glomerular filtration rate (GFR) is in mL/min/1.73 m^2^ body surface area. The product of this equation for women was multiplied by a correction factor of 0.739. Serum creatinine was measured by enzymatic methods in the same laboratory. Random urine samples (10 mL) were also collected at baseline to measure the ratio of urinary protein to creatinine. Other baseline variables included age, sex, diabetes defined according to the International Classification of Diseases, Tenth Revision (ICD-10) codes E10-E14, systolic blood pressure, diastolic blood pressure, hemoglobin, albumin, calcium, phosphate, and use of renin-angiotensin system inhibitor, beta-blocker, and calcium blocker. Cardiovascular disease included ischemic heart disease (ICD-10 codes I20-I25), heart failure (ICD-10 code I50), and stroke (ICD-10 codes I60-I67).

The primary end points were end-stage kidney disease (ESKD) requiring kidney replacement therapy and a composite of ESKD and all-cause of death.

Patients received regular follow-up care in the outpatient ward. Data were collected from source documentation at the end of 2011. The baseline and follow-up data were collected from hospital medical records and discharge abstracts, outpatient visit records, contact with primary and dialysis care physicians and death certificates. End points were validated by at least two physicians. The follow-up of patients is available with accuracy because i) this facility is the central hospital of the southern area of Osaka prefecture, and there is no other central hospital in this region, and ii) regional partnership with the primary and dialysis care physicians has been well-established.

### Metabolite extraction and measurement

Metabolites were measured as previously reported with modification^21^,^37^. Briefly, plasma samples (40 μl) were put into 360 μl of methanol which contained internal standards [20 μmol/L each of methionine sulfone and 2-(N-morpholino)-ethanesul-fonic acid]. The homogenate was then mixed with 160 μl of Milli-Q water and 400 μl chloroform and centrifuged at 10,000 g for 5 min at 4 °C. Subsequently, the aqueous 300 μl solution was centrifugally filtered through a 5-kDa cutoff filter (Millipore) to remove proteins. The filtrate was centrifugally concentrated and dissolved in 50 μl Milli-Q water that contained reference compounds (200 μmol/L each of 3-aminopyrrolidine and trimesate) immediately before CE-TOFMS analysis.

Measurement of metabolites was performed using CE-TOMFS^21^,^37^. Briefly, CE-TOFMS was carried out using an Agilent CE Capillary Electrophoresis System equipped with 6210 Time-of-Flight mass spectrometer, 1100 isocratic HPLC pump, G1603A CE-MS adapter kit, and G1607A CE-ESI-MS sprayer kit. The system was controlled by Agilent G2201AA ChemStation software version B.03.01 for CE. Data acquisition was performed by Analyst QS Build: 7222 software for Agilent TOF (Applied Biosystems). For cationic metabolites, capillary electrophoreses were performed using a fused silica capillary. The electrolyte was 1 M formic acid. For anionic metabolites, a polymer coated COSMO (+) capillary (Nacalai, 07584-44i) was used. The electrolyte was 50 mM ammonium acetate (pH 8.5). For all analytical modes, inner diameter and total length of capillary are 50 μm and 100 cm, respectively. The applied voltage was set at +30 kV and −30 kV for cation and anion modes and nucleotide mode, respectively. Electrosplay ionisation-TOFMS was operated in the positive ion mode (4 kV), the negative ion mode (3.5 kV), and the negative ion mode (3.5 kV) for cationic metabolites, anionic metabolites, and nucleotides, respectively. Exact mass data were acquired over a 50–1000 *m/z* range. A mixed solution of the standards were prepared immediately before CE-TOFMS analysis as previously described^22^. The measured metabolite included amino acids, glycolysis intermediates, nucleotides, and their derivatives. CE exhibits extremely high resolution for charged species and its injection volume is quite low (3–30 nl). Therefore, matrix effects are hardly observed in CE-MS system^38^,^39^.

### Data processing

Raw data were processed using software (MasterHands) developed in-house as previously described^22^. The data processing flow consisted of noise filtering, baseline correction, peak detection, and integration of the peak areas from 0.02 m/z-wide sections of the electropherograms. The accurate m/z of each peak was calculated by Gaussian curve-fitting on the m/z domain, and the migration times were normalized to match the detected peaks among the multiple datasets. The peaks were identified by matching m/z values and normalized migration times of corresponding authentic standard compounds. Quantification was performed by comparing the peak areas against a calibration curves generated using internal standardization-techniques to eliminate systematic bias which was derived from injection volume variance and MS sensitivity. Metabolites that were above the detection limit in at least 40% of study subjects were analyzed. We set this cut-off value for two reasons. First, we were afraid higher cut-off value may lead us to underestimate the effects of metabolites, which is against the screening-nature of this study. Second, we consider the possibility that the detection of a certain metabolite in itself may provide a valuable information for the prognosis. The levels of metabolites under detection limits were imputed with minimal value of each metabolite. The levels of metabolites collected here were exposed to the Holm’s multiple comparison as described below.

### Statistical analysis

We constructed multivariate Cox proportional hazard models to assess an association between each candidate metabolite and the outcome after adjusting for the baseline covariates. Multiple comparison of the hazard models were performed with the Holm method. Log-rank test was used to assess the equality of survival distribution stratified by the median values. Statistical significance was defined as *P* < 0.05. Statistical analyses were performed using R environment for statistical computing, version 2.15.2^40^, and STATA statistical software version 11 (STATA Corporation, College Station, TX, USA).

## Additional Information

**How to cite this article**: Kimura, T. *et al.* Identification of biomarkers for development of end-stage kidney disease in chronic kidney disease by metabolomic profiling. *Sci. Rep.* **6**, 26138; doi: 10.1038/srep26138 (2016).

## Supplementary Material

Supplementary Information

## Figures and Tables

**Table 1:** Baseline characteristics of the patients.

Characteristic	Patients (n = 112)
Age (yr)	65.3 ± 10.9
Male gender (%)	75.0
eGFR (mL/min/1.73 m^2^)	21.1 ± 12.4
Mean blood pressure (mmHg)	94.9 ± 13.0
Systolic blood pressure (mmHg)	138.7 ± 22.1
Diastolic blood pressure (mmHg)	73.0 ± 11.6
History of cardiovascular disease (%)	35.7
Hemoglobin (g/dL)	11.0 ± 1.9
Urinary protein (g/gCre)	2.7 ± 3.7
Origin of disease (%)	
Diabetes mellitus	31.2
Chronic glomerular nephritis	24.1
Others	44.7
Use of ACEi and/or ARB (%)	68.8
Use of beta-blocker (%)	32.3
Use of calcium blocker (%)	63.4

Values are described as mean ± SD, or %. eGFR, estimated glomerular filtration ratio; ACEi, angiotensin converting enzyme inhibitor; ARB, angiotensin receptor blocker.

**Table 2:** Cox regression analysis of the effect of plasma metabolites on the risk of composite outcome.

Metabolite	Tertile as a continuous variable		*P*	2nd		*P*	3rd		*P*
Isethionate	2.92	(1.76–4.84)	<0.001	2.38	(1.02–5.54)	0.044	8.04	(2.92–22.14)	<0.001
Saccharate	2.60	(1.65–4.10)	<0.001	3.20	(1.33–7.65)	0.009	7.32	(2.76–19.40)	<0.001
Trimethylamine N-oxide	2.30	(1.54–3.43)	<0.001	2.57	(1.08–5.66)	0.032	5.41	(2.33–12.56)	<0.001
4-Oxopentanoate	1.73	(1.17–2.56)	0.006	2.34	(1.03–5.32)	0.043	3.35	(1.43–7.86)	0.006
Cytidine	1.96	(1.33–2.89)	0.001	2.04	(0.92–4.53)	0.079	3.86	(1.76–8.46)	0.001
Gluconate	2.50	(1.41–4.44)	0.002	1.54	(0.67–3.55)	0.31	5.68	(1.89–17.09)	0.002
Glucuronate	2.14	(1.32–3.46)	0.002	2.10	(0.90–4.90)	0.087	4.53	(1.68–12.26)	0.003
Guanidinosuccinate	2.19	(1.36–3.54)	0.001	1.70	(0.66–4.40)	0.28	4.19	(1.49–11.83)	0.007
2-Hydroxyisobutyrate	1.76	(1.16–2.69)	0.008	1.19	(0.55–2.56)	0.66	2.84	(1.25–6.49)	0.013
Uridine	2.00	(1.19–3.34)	0.008	1.43	(0.60–3.39)	0.42	3.62	(1.29–10.14)	0.014
5-Oxoproline	1.56	(1.08–2.25)	0.019	1.48	(0.69–3.16)	0.31	2.39	(1.12–5.08)	0.024
Pimelate	1.43	(0.98–2.08)	0.065	2.38	(1.03–5.46)	0.042	2.51	(1.08–5.82)	0.032
N-Acetylneuraminate	1.62	(1.04–2.53)	0.032	1.74	(0.84–3.62)	0.14	2.64	(1.08–6.44)	0.033
3-Methylhistidine	1.61	(1.03–2.50)	0.035	1.62	(0.71–3.69)	0.25	2.59	(1.05–6.41)	0.040
Phthalate	1.46	(1.02–2.10)	0.041	0.74	(0.35–1.56)	0.42	2.01	(1.02–3.96)	0.043
Citramalate	1.63	(1.03–2.58)	0.036	0.70	(0.33–1.49)	0.36	2.42	(1.01–5.82)	0.047
Trigonelline	1.19	(0.84–1.70)	0.33	2.31	(1.09–4.88)	0.029	1.62	(0.75–3.51)	0.22
Trp	0.69	(0.46–1.03)	0.068	0.72	(0.39–1.32)	0.29	0.46	(0.20–1.06)	0.069
2,3-Pyridinedicarboxylate	1.54	(0.99–2.39)	0.054	1.30	(0.68–2.93)	0.52	2.24	(0.91–5.51)	0.079
Choline	1.38	(0.95–2.01)	0.094	1.64	(0.73–3.69)	0.23	2.01	(0.90–4.48)	0.09
Hippurate	1.49	(1.01–2.20)	0.045	0.74	(0.34–1.61)	0.45	1.89	(0.90–3.97)	0.092
trans-Aconitate	1.41	(0.93–2.16)	0.11	1.20	(0.57–2.53)	0.64	1.93	(0.83–4.49)	0.13
3-Hydroxy-3-methylglutarate	1.11	(0.76–1.63)	0.6	1.74	(0.83–3.65)	0.14	1.25	(0.58–2.70)	0.57
Isocitrate	1.15	(0.77–1.70)	0.49	0.73	(0.33–1.60)	0.43	1.14	(0.52–2.50)	0.74

Data are hazard ratios (95% confidence interval) estimated as the effect per one tertile as continuous variable or each tertile as categorical variable (first tertile was used as reference) of the metabolite. Models were developed by adjustment for eGFR, the level of urinary protein, the presence of diabetes, age, sex, calcium*phosphate, mean blood pressure, the presence of past cardiovascular events, and the level of hemoglobin.
