# Supplementary material for: Identification of biomarkers for development of end-stage kidney disease in chronic kidney disease by metabolomic profiling
Source: Sci Rep. 2016 May 18;6:26138. doi: 10.1038/srep26138 (PMC4870629; doi:10.1038/srep26138)
Supplement: Supplementary Information [file srep26138-s1.pdf]

Supplemental Information

Identification of biomarkers for development of end-stage kidney disease in chronic kidney disease by metabolomic profiling

Tomonori Kimura, Keiko Yasuda, Ryohei Yamamoto, Keiko Yasuda, Tomoyoshi Soga, Hiromi Rakugi, Terumasa Hayashi, and Yoshitaka Isaka

5 Supplemental figures
3 Supplemental tables

Figure S1

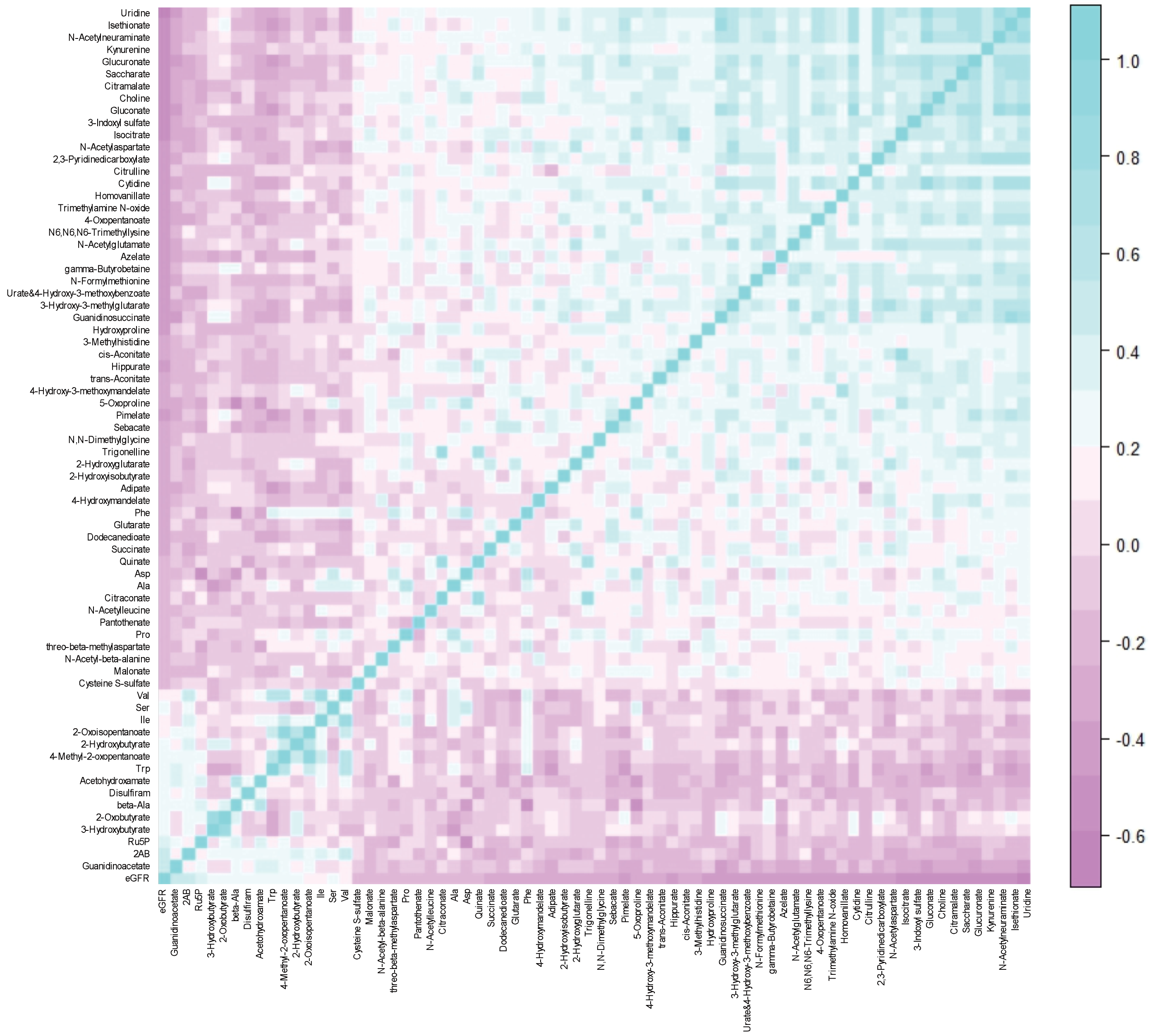

Figure S1. Correlation matrix for serum metabolite levels with estimated glomerular filtratior ratio.

Figure S2

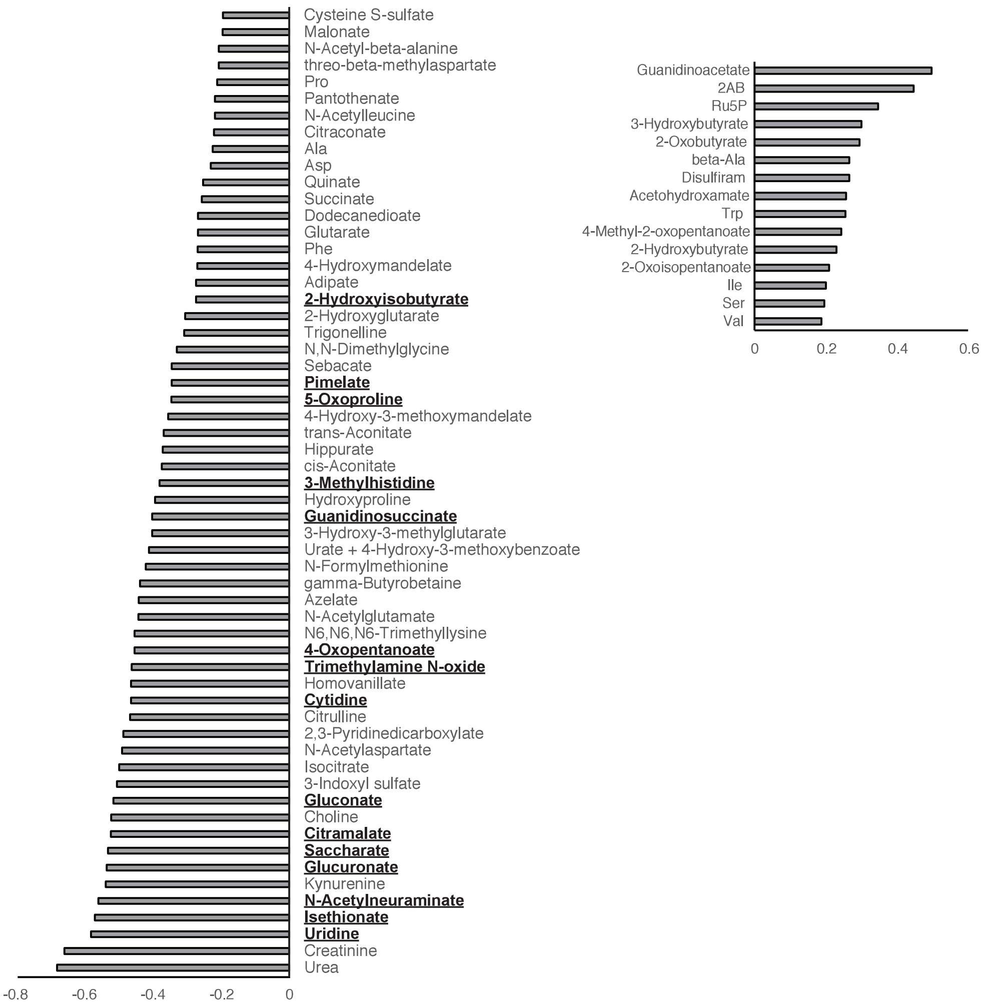

Figure S2. Spearman correlation coefficients betweeneGFR and each metabolite.The name of the metabolites selected as the risk for the kidney outcome in the prediction set are highlighted by bold letters with underlines.

Figure S3

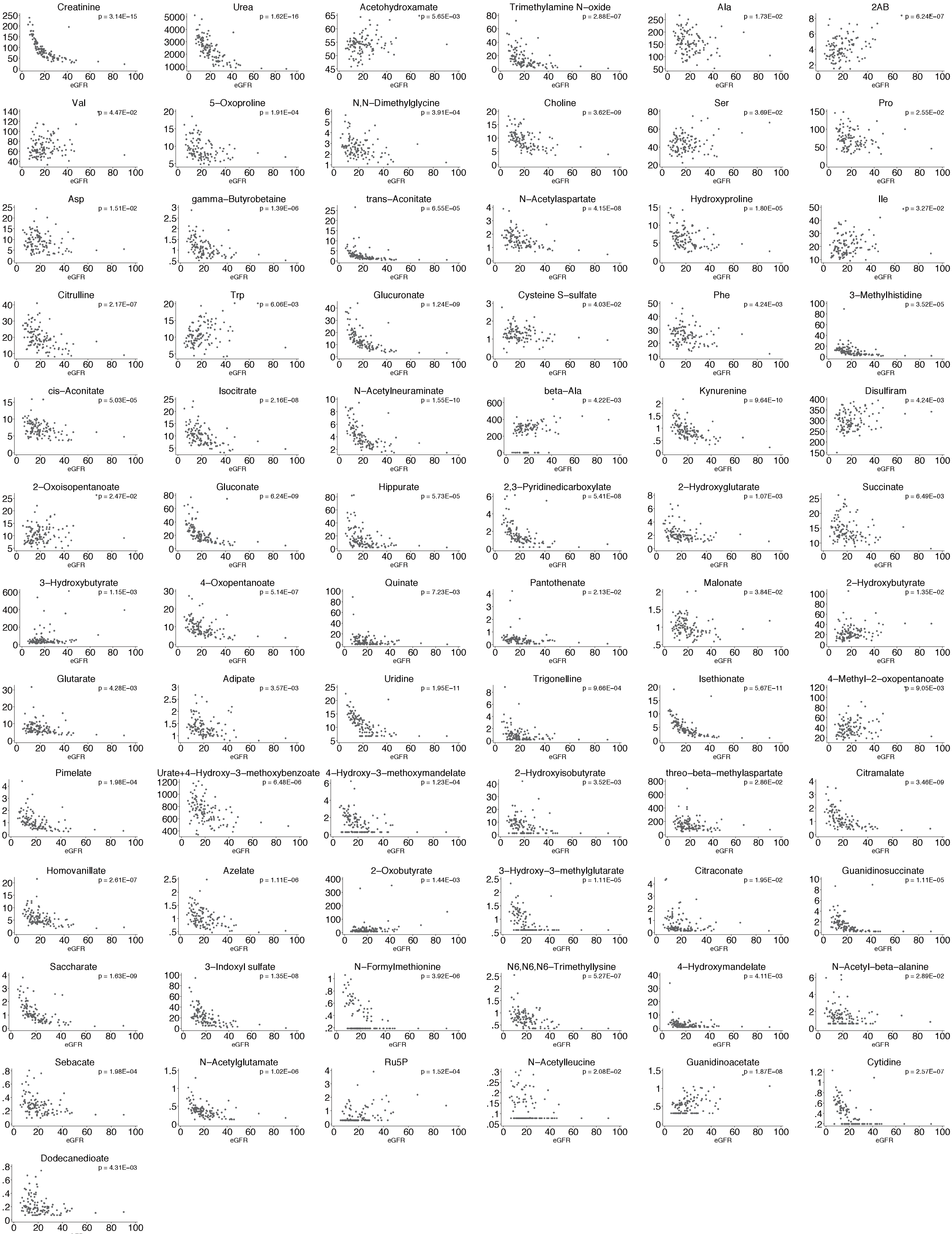

Figure S3. Scatter plots of eGFR with the levels of metabolites which were correlated in this study. The units for metabolites and eGFR are μM and mL/min/1.73m^2, respectively.

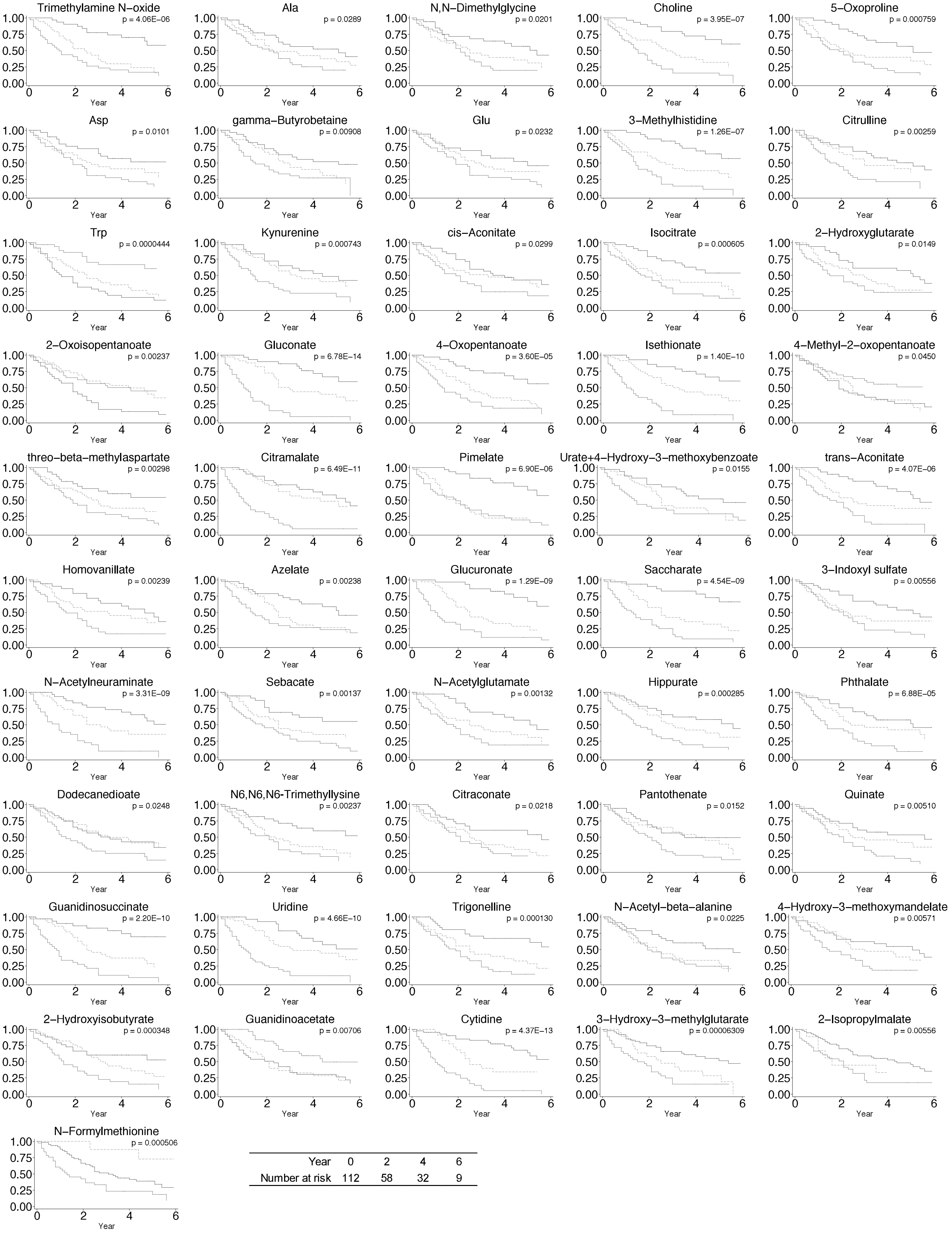

Year	0	2	4	6
Number at risk	112	58	32	9

Figure S4. Kaplan-Meier survival curves of metabolites which significantly predicted prognosis of the prognosis of composite outcome Patients with first (thick line), second (dotted line), and third (thin gray line) tertile of levels of metabolites were subjected to these analyses.

A

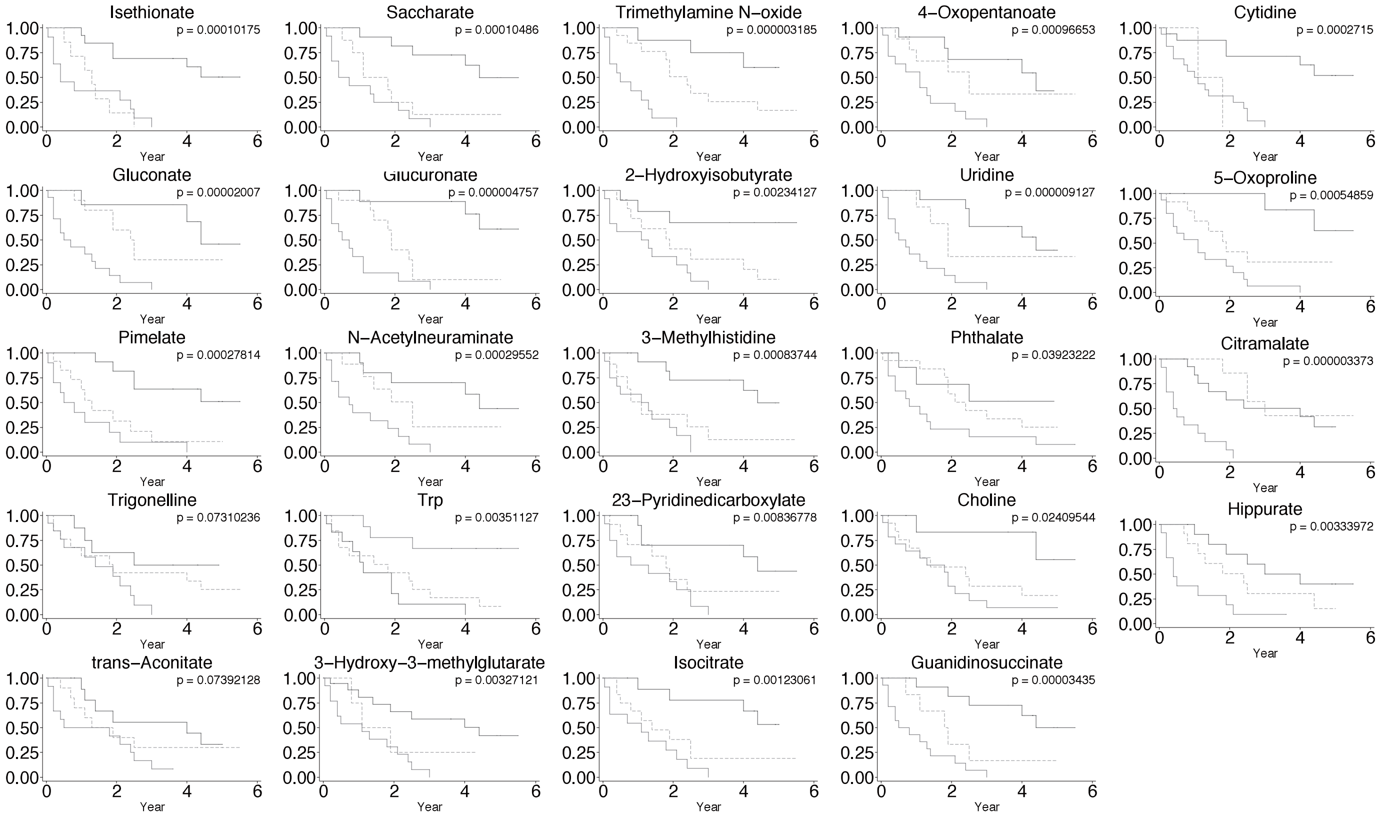

B

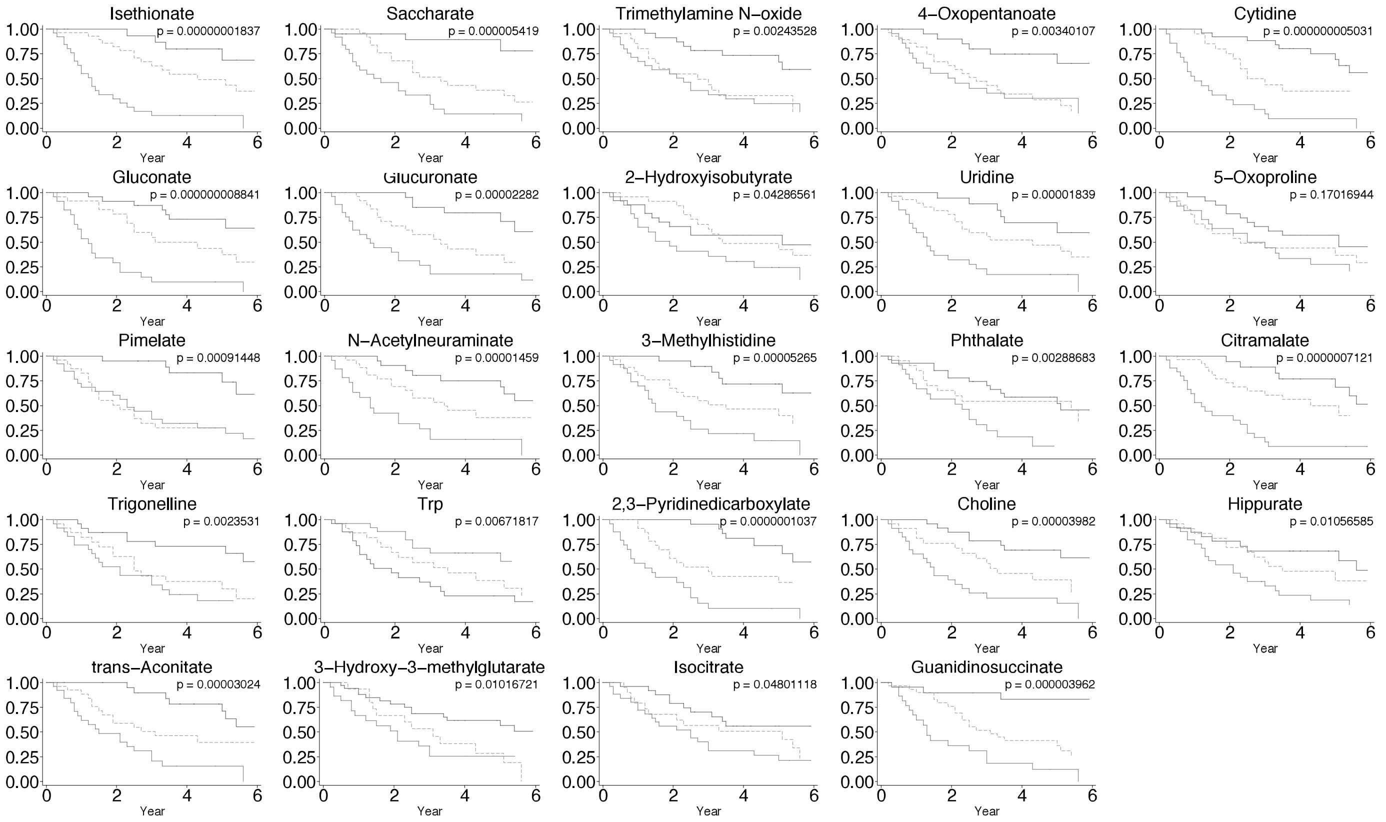

Number at risk

Year	0	2	4	6
Diabetic patients	35	14	8	1
Non-diabetic patients	77	44	24	8

Figure S5. Kaplan-Meier survival curves of selected metabolites for the composite outcome stratified (A) in the presene or (B) absence of diabetes. Patients with first (thick line), second (dotted line), and third (thin gray line) tertile of levels of metabolites were subjected to these analyses.

Table S1. Holm-adjusted Cox regression analyses for the progression of kidney disease

Compond Name	Unadjusted *P*	Adjusted *P*
Gluconate	2.4E-08	3.1E-06
Cytidine	1.8E-07	2.2E-05
N-Acetylneuraminate	4.8E-07	6.1E-05
2,3-Pyridinedicarboxylate	2.0E-06	2.6E-04
Uridine	2.3E-06	3.0E-04
Guanidinosuccinate	3.1E-06	3.9E-04
Isethionate	3.6E-06	4.6E-04
Choline	5.2E-06	6.6E-04
Citramalate	6.2E-06	7.8E-04
3-Methylhistidine	1.4E-05	1.8E-03
3-Hydroxy-3-methylglutarate	1.4E-05	1.8E-03
Saccharate	2.4E-05	3.1E-03
Pimelate	2.6E-05	3.2E-03
5-Oxoproline	3.0E-05	3.8E-03
Glucuronate	3.1E-05	4.0E-03
Trimethylamine N-oxide	4.8E-05	6.1E-03
trans-Aconitate	1.2E-04	0.015
Trigonelline	4.1E-04	0.05
4-Oxopentanoate	4.7E-04	0.06
Isocitrate	7.7E-04	0.10
Kynurenine	1.5E-03	0.20
Trp	1.9E-03	0.23
Phthalate	2.0E-03	0.25
2-Isopropylmalate	2.3E-03	0.30
Sebacate	2.5E-03	0.32
threo-beta-methylaspartate	2.7E-03	0.34
N-Acetylglutamate	3.1E-03	0.39
Thymine	3.3E-03	0.42
Ala	3.5E-03	0.45
2-Hydroxyisobutyrate	4.3E-03	0.55
2-Hydroxyglutarate	5.2E-03	0.66
N,N-Dimethylglycine	5.8E-03	0.74
Citraconate	6.8E-03	0.86
Homovanillate	7.1E-03	0.9
Guanidinoacetate	7.8E-03	1
Dodecanedioate	8.0E-03	1
gamma-Butyrobetaine	8.6E-03	1
4-Hydroxy-3-methoxymandelate	0.013	1
N-Acetyl-beta-alanine	0.013	1
Quinate	0.014	1
Allantoin	0.015	1

Azelate	0.017	1
Hippurate	0.019	1
Citrulline	0.023	1
Asp	0.027	1
Malonate	0.031	1
4-Hydroxymandelate	0.036	1
Pantothenate	0.037	1
2-Oxoisopentanoate	0.037	1
Hydroxyproline	0.042	1
Glu	0.046	1
N-Acetylleucine	0.050	1
3-Indoxyl sulfate	0.052	1
Urate + 4-Hydroxy-3-methoxybenzoate	0.055	1
Betaine	0.059	1
Undecanoate	0.063	1
cis-Aconitate	0.068	1
Pro	0.076	1
N-Formylmethionine	0.096	1
N6,N6,N6-Trimethyllysine	0.10	1
Phe	0.10	1
alpha-Aminoadipate	0.10	1
Taurine	0.11	1
4-Pyridoxate	0.13	1
N-Acetylaspartate	0.13	1
Cholate	0.15	1
Octanoate	0.15	1
Syringate	0.16	1
Ornithine	0.16	1
Decanoate	0.17	1
Glutarate	0.18	1
Citrate	0.18	1
Cysteine S-sulfate	0.18	1
beta-Ala	0.19	1
Adipate	0.21	1
Creatine	0.22	1
Succinate	0.22	1
Gly-Gly	0.22	1
Gly	0.22	1
Met	0.24	1
Ru5P	0.25	1
4-Acetylbutyrate	0.29	1
2-Oxobutyrate	0.30	1
Fumarate	0.31	1
Cyclohexylamine	0.31	1
o-Acetylcarnitine	0.34	1

4-Methyl-2-oxopentanoate	0.34	1
Dihydrouracil	0.35	1
Diethanolamine	0.36	1
Terephthalate	0.37	1
Lys	0.40	1
Ser	0.41	1
Sarcosine	0.42	1
Cystine	0.44	1
Dodecanoate	0.45	1
2-Hydroxybutyrate	0.46	1
Asn	0.47	1
Val	0.48	1
Lactate	0.48	1
Pelargonate	0.50	1
Glycerophosphate	0.50	1
Tyr	0.52	1
2AB	0.52	1
His	0.53	1
2-Oxooctanoate	0.55	1
Malate	0.56	1
Carnitine	0.56	1
Hexanoate	0.58	1
Glycocholate	0.59	1
Acetohydroxamate	0.61	1
Ile	0.64	1
2-Hydroxypentanoate	0.64	1
6-Aminopenicillanate	0.65	1
Heptanoate	0.66	1
Pipecolate	0.67	1
2-Oxoglutarate	0.69	1
Disulfiram	0.70	1
2-Hydroxy-4-methylpentanoate	0.74	1
Hypoxanthine	0.75	1
3-Hydroxybutyrate	0.79	1
Leu	0.79	1
Arg	0.86	1
Methionine sulfoxide	0.87	1
Thr	0.89	1
Gln	0.93	1
Mucate	0.94	1
1-Methyl-2-pyrrolidinone	0.98	1

Crude *P* values for trend were determined by Cox regression analyses. Adjustment for crude *P* values were performed by Holm-method (adjusted *P*).

Supplementary Table 2. Cox regression analysis of the effect of plasma metabolites on the risk of progression to ESKD.

Metabolite	Tertile as a continuous variable		*P*	2nd		*P*	3rd		*P*
Isethionate	3.32	(1.94-5.68)	<0.001	2.80	(1.12-7.04)	0.028	10.4	(3.50-30.85)	< 0.001
Saccharate	2.71	(1.68-4.39)	<0.001	3.17	(1.25-8.04)	0.015	7.87	(2.82-21.98)	< 0.001
Trimethylamine N-oxide	2.33	(1.54-3.56)	<0.001	2.82	(1.16-6.86)	0.022	5.85	(2.37-14.44)	< 0.001
4-Oxopentanoate	1.69	(1.12-2.55)	0.012	2.29	(0.96-5.47)	0.062	3.24	(1.32-7.92)	0.010
Cytidine	2.10	(1.40-3.15)	<0.001	2.02	(0.86-4.73)	0.11	4.39	(1.94-9.94)	< 0.001
Gluconate	2.91	(1.60-5.29)	<0.001	1.79	(0.73-4.43)	0.21	7.38	(2.32-23.44)	0.001
Glucuronate	2.08	(1.26-3.43)	0.004	2.04	(0.83-5.00)	0.12	4.29	(1.51-12.16)	0.006
Guanidinosuccinate	2.35	(1.42-3.87)	0.001	1.69	(0.62-4.63)	0.31	4.59	(1.54-13.68)	0.006
2-Hydroxyisobutyrate	1.77	(1.14-2.75)	0.011	1.06	(0.47-2.38)	0.89	2.75	(1.17-6.46)	0.020
Uridine	2.21	(1.29-3.79)	0.004	1.52	(0.61-3.82)	0.37	4.31	(1.46-12.75)	0.008
5-Oxoproline	1.44	(0.98-2.11)	0.065	1.31	(0.60-2.85)	0.50	2.01	(0.92-4.39)	0.079
Pimelate	1.25	(0.84-1.87)	0.28	2.06	(0.88-4.87)	0.098	1.92	(0.79-4.65)	0.149
N-Acetylneuraminate	1.89	(1.18-3.01)	0.008	2.27	(1.02-5.03)	0.044	3.61	(1.39-9.38)	0.009

3-Methylhistidine	1.60	(1.01-2.53)	0.045	1.55	(0.65-3.66)	0.32	2.53	(0.98-6.50)	0.054
Phthalate	1.40	(0.96-2.04)	0.081	0.56	(0.25-1.23)	0.15	1.83	(0.92-3.66)	0.086
Citramalate	1.97	(1.21-3.20)	0.006	0.86	(0.39-1.91)	0.71	3.35	(1.33-8.43)	0.010
Trigonelline	1.22	(0.85-1.76)	0.28	3.05	(1.36-6.82)	0.007	1.83	(0.80-4.19)	0.15
Trp	0.72	(0.47-1.10)	0.13	0.77	(0.41-1.44)	0.41	0.51	(0.21-1.21)	0.13
2,3-Pyridinedicarboxylate	1.61	(1.02-2.55)	0.042	1.45	(0.61-3.43)	0.40	2.49	(0.96-6.45)	0.060
Choline	1.33	(0.90-1.97)	0.151	1.38	(0.60-3.18)	0.44	1.79	(0.80-4.05)	0.16
Hippurate	1.51	(1.01-2.26)	0.046	0.91	(0.41-2.04)	0.83	2.00	(0.91-4.4)	0.083
trans-Aconitate	1.45	(0.93-2.26)	0.11	1.35	(0.61-2.97)	0.46	2.07	(0.85-5.05)	0.11
3-Hydroxy-3-methylglutarate	1.11	(0.74-1.64)	0.63	1.40	(0.63-3.10)	0.41	1.23	(0.55-2.74)	0.61
Isocitrate	1.06	(0.71-1.60)	0.76	0.59	(0.26-1.32)	0.20	0.95	(0.42-2.11)	0.90

Data are hazard ratios (95% confidence interval) estimated as the effect per one tertile as continuous variable or each tertile as categorical variable (first tertile was used as reference) of the metabolite. Models were developed by adjustment for eGFR, the level of urinary protein, the presence of diabetes, age, sex, calcium*phosphate, mean blood pressure, the presence of past cardiovascular events, and the level of hemoglobin.

Supplementary Table 3. Cox regression analysis of the effect of plasma metabolites on the risk of composite outcome stratified by the presence of diabetes.

Metabolite	Diabetic patients		*P*	Non-diabetic patients		*P*
Isethionate	4.70	(1.79-12.32)	0.002	3.42	(1.64-7.12)	0.001
Saccharate	4.35	(1.79-12.32)	0.001	2.53	(1.35-4.75)	0.004
Trimethylamine N-oxide	6.59	(2.40-18.05)	<0.001	2.01	(1.25-3.22)	0.004
4-Oxopentanoate	4.64	(1.67-12.91)	0.003	1.56	(0.92-2.62)	0.10
Cytidine	3.67	(1.60-8.41)	0.002	2.33	(1.26-4.32)	0.007
Gluconate	10.27	(2.31-45.72)	0.002	2.25	(1.11-4.56)	0.024
Glucuronate	8.17	(2.73-24.47)	<0.001	1.79	(0.98-3.23)	0.058
Guanidinosuccinate	3.79	(1.52-9.48)	0.004	1.92	(1.04-3.56)	0.037
2-Hydroxyisobutyrate	3.95	(1.50-10.39)	0.005	1.12	(0.64-1.98)	0.69
Uridine	4.49	(1.48-13.56)	0.008	2.05	(1.05-4.01)	0.036
5-Oxoproline	5.08	(2.12-12.18)	<0.001	1.40	(0.90-2.18)	0.130
Pimelate	2.23	(1.15-4.31)	0.017	1.09	(0.64-1.88)	0.740

N-Acetylneuraminate	3.09	(1.24-7.68)	0.015	1.35	(0.79-2.31)	0.27
3-Methylhistidine	1.98	(1.04-3.60)	0.038	1.43	(0.79-2.57)	0.24
Phthalate	2.95	(1.32-6.63)	0.009	0.97	(0.62-1.50)	0.88
Citramalate	1.92	(0.79-4.68)	0.150	2.38	(1.28-4.45)	0.006
Trigonelline	0.90	(0.40-2.00)	0.790	1.29	(0.84-1.96)	0.25
Trp	0.43	(0.18-0.99)	0.047	0.79	(0.49-1.28)	0.35
2,3-Pyridinedicarboxylate	1.54	(0.70-3.37)	0.280	2.21	(1.22-4.03)	0.009
Choline	1.06	(0.52-2.18)	0.870	1.38	(0.88-2.16)	0.16
Hippurate	2.03	(1.07-3.86)	0.030	1.36	(0.85-2.17)	0.20
trans-Aconitate	1.06	(0.48-2.36)	0.880	1.65	(1.00-2.72)	0.050
3-Hydroxy-3-methylglutarate	2.13	(1.06-4.29)	0.034	0.73	(0.45-1.19)	0.21
Isocitrate	2.10	(0.82-5.37)	0.120	1.22	(0.73-2.02)	0.44

Data are hazard ratios (95% confidence interval) estimated as the effect per one tertile as continuous variable of the metabolite. Models were developed by adjustment for eGFR, the level of urinary protein, age, sex, calcium*phosphate, mean blood pressure, the presence of past cardiovascular events, and the level of hemoglobin.
